# Supplementary material for: Pan-cancer screen for mutations in non-coding elements with conservation and cancer specificity reveals correlations with expression and survival
Source: NPJ Genom Med. 2018 Jan 11;3:1. doi: 10.1038/s41525-017-0040-5 (PMC5765157; doi:10.1038/s41525-017-0040-5)
Supplement: Supplementary file 1 — Supplementary Information [file 41525_2017_40_MOESM1_ESM.pdf]

Supplementary Information for:

Pan-cancer screen for mutations in non-coding elements with conservation and cancer specificity reveals correlations with expression and survival

Henrik Hornshøj*[1,4], Morten Muhlig Nielsen[1,4], Nicholas A. Sinnott-Armstrong[3], Michał P. Świtnicki[1], Malene Juul[1], Tobias Madsen[1,2], Richard Sallari[3], Manolis Kellis[3], Torben Ørntoft[1], Asger Hobolth[2] and Jakob Skou Pedersen*[1,2]

1) Department of Molecular Medicine, Aarhus University Hospital, Palle Juul-Jensens Boulevard 99, 8200 Aarhus, Denmark. 2) Bioinformatics Research Centre, Aarhus University, C.F. Møllers Allé 8, 8000 Aarhus C, Denmark. 3) Computer Science and Artificial Intelligence Laboratory, Massachusetts Institute of Technology, Cambridge, Massachusetts 02140, USA. 4) Contributed equally.

Running title: Pan-cancer non-coding drivers, expression correlation and survival
Keywords: pan-cancer, regulatory drivers, expression correlation, survival

*) Corresponding authors:
Jakob Skou Pedersen (jakob.skou@clin.au.dk)
Henrik Hornshøj (hhj@clin.au.dk)

Department of Molecular Medicine (MOMA)
Aarhus University Hospital
Palle Juul-Jensens Boulevard 99
8200 Aarhus N, Denmark

Supplementary Figures

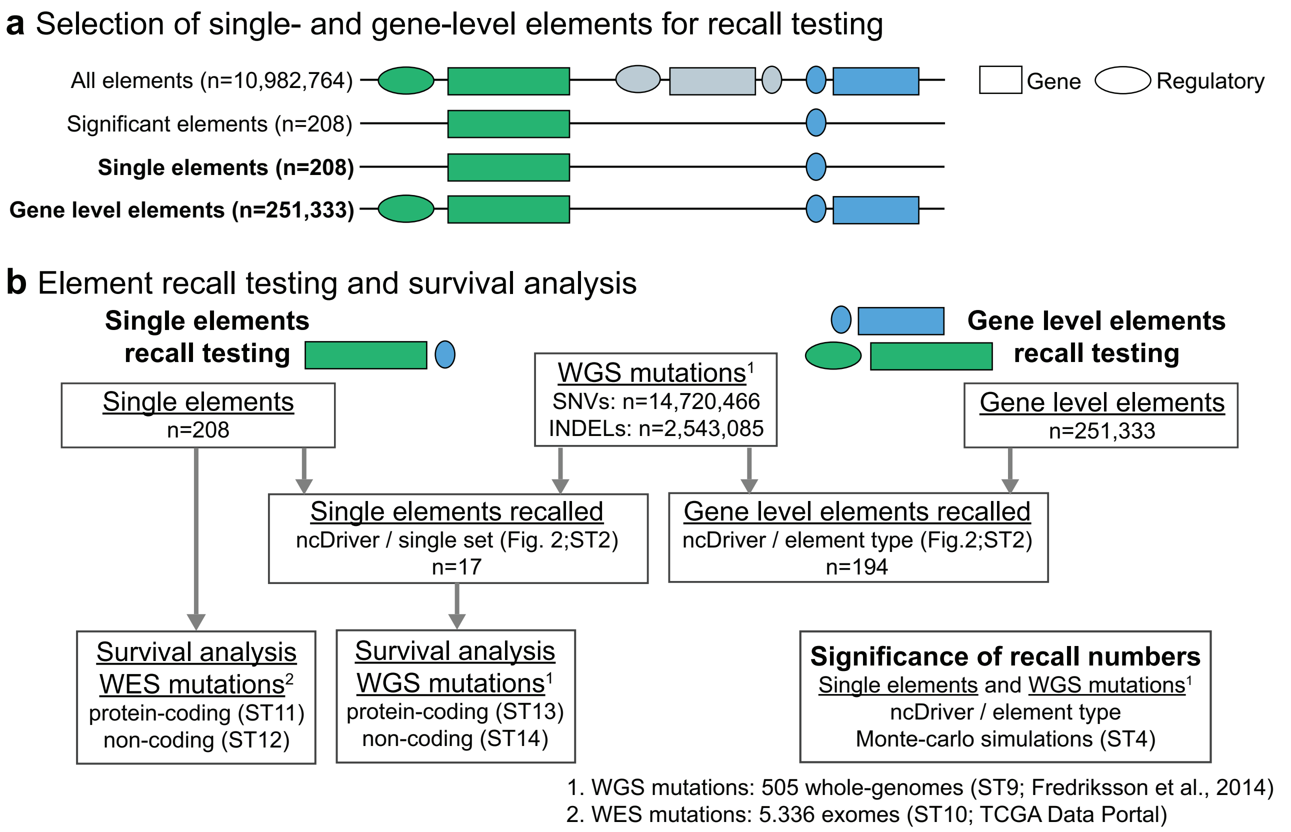

Supplementary Figure 1: Recall procedure for significant elements and survival analysis.
(**a**) Illustration showing how single and gene-level elements were selected for recall testing based on 208 significant elements. Single elements for recall testing were a direct transfer of the candidate elements, whereas the gene-level elements represented an extended element sets including all elements sharing the same gene IDs as the candidate elements. Elements belonging to the same gene are indicated with the same color code. (**b**) Workflow chart showing the elements and mutations used as input for recall testing, and the resulting recalled elements. Where appropriate, references are given in brackets to figures and tables with corresponding results.

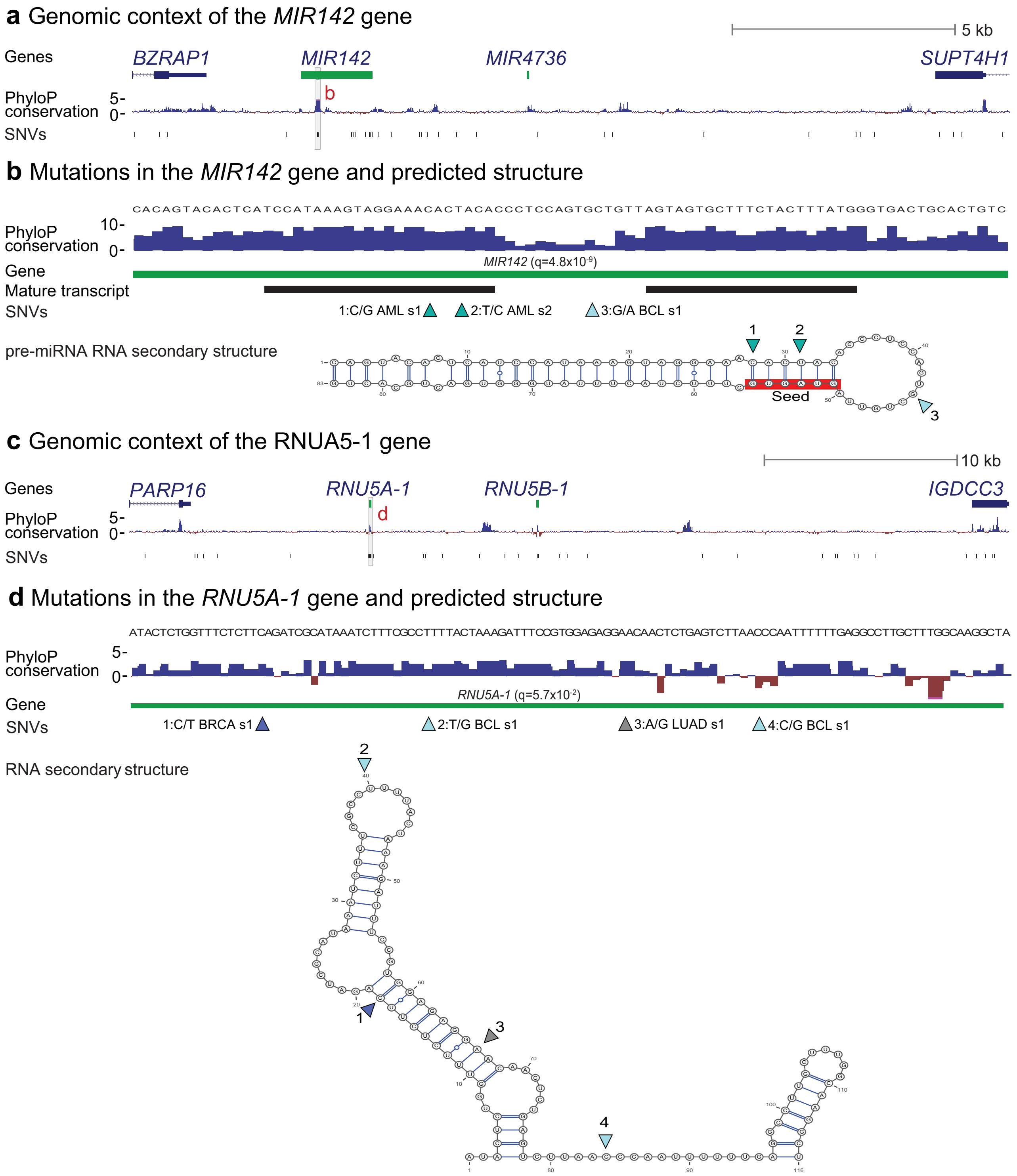

Supplementary Figure 2: *MIR142* and *RNU5A-1* identified as significant ncRNAs.
(**a**) Genomic context of the *MIR142* gene. (**b**) Primary sequence, PhyloP positional conservation scores, and location of the mature miRNA and its star form are shown together with SNV hits for the precursor miRNA region. Below, the EvoFold prediction[1,2] of the RNA secondary structure is given with indication of miRNA seed site (red bar) and SNV locations (enumerated triangles). (**c**) Genomic context of the *RNU5A-1* gene. (**d**) Primary sequence, PhyloP positional conservation scores, and SNVs for the *RNU5A-1* locus. Below, EvoFold RNA secondary structure prediction[1,2] with indication of SNV locations.

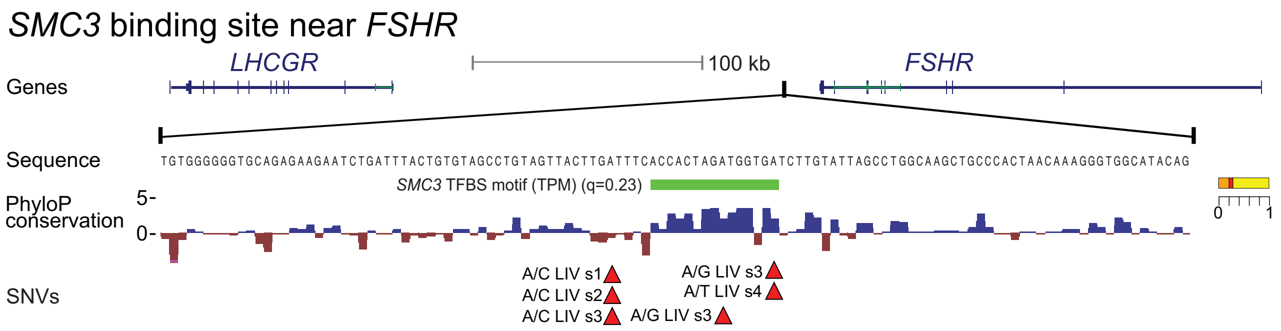

Supplementary Figure 3: Significant *SMC3* TFBS near *FSHR*.
Top rows show the genomic context with nearby gene and rows below show detailed views of primary sequence, the regulatory elements, PhyloP conservation scores and SNVs. SNV annotation and relative significance contribution of mutational distribution tests as in **Fig 2a and 3**.

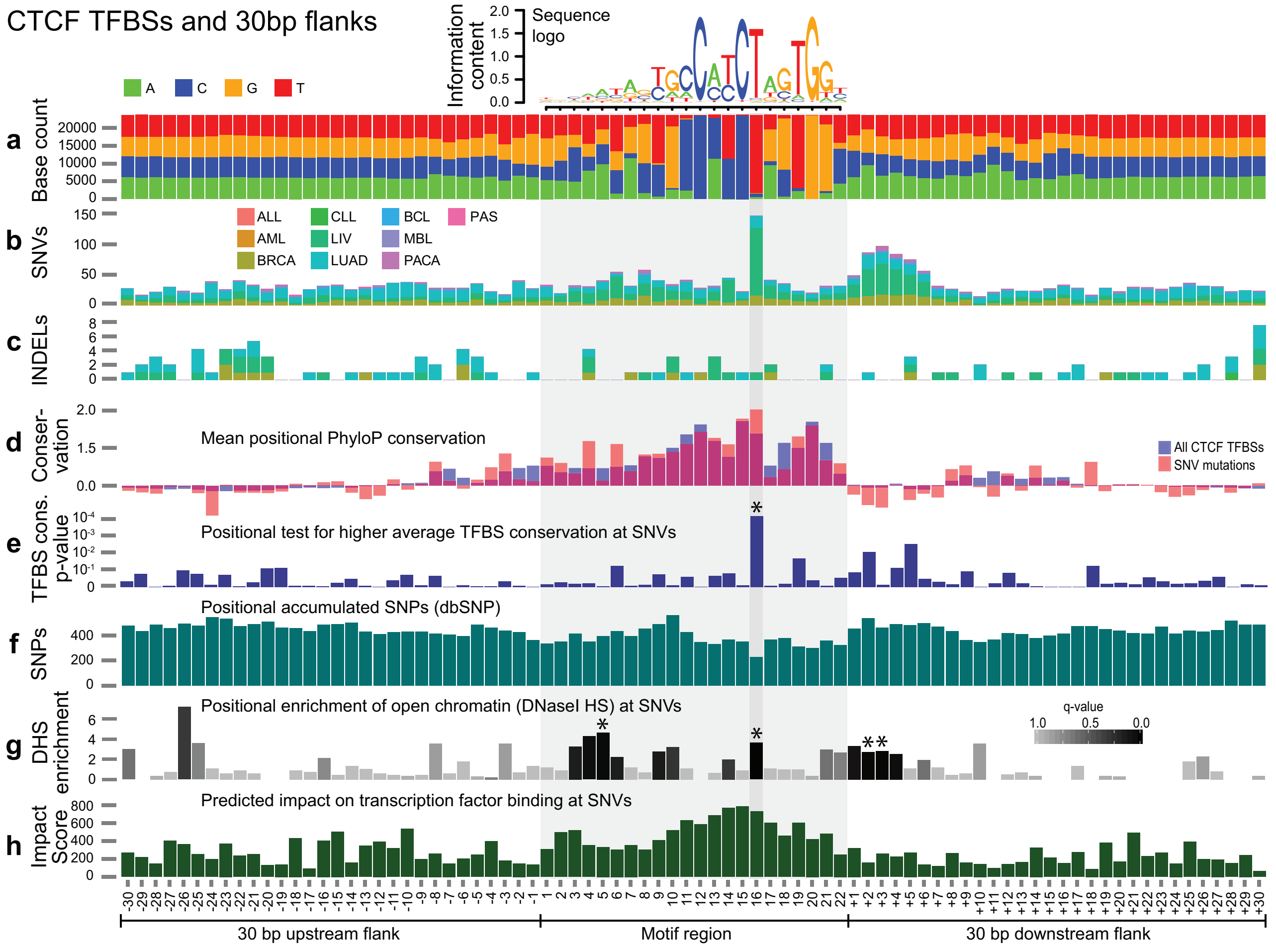

Supplementary Figure 4: Genome-wide mutation analysis of *CTCF* TFBSs and 30bp flanking regions.
Sequence logo and positional characterization of mutations in *CTCF* TFBSs (subtype disc1) and their 30bp flanking regions accumulated across the genome. (**a**) *Base count* shows the nucleotide composition across all *CTCF* TFBSs. (**b**,**c**) The positional distributions of *SNVs* and *INDELs* have cancer type contributions shown with colors (see caption). (**d**) The mean positional phyloP *conservation* is shown for both the background (all *CTCF* TFBSs; blue) and positions hit by SNVs (red). (**e**) The correlation between presence of SNV and high average conservation of the associated *CTCF* TFBS is evaluated per position and denoted by the *Conservation test p-value* (**Supplemental Fig. 5**). Position 16 shows a significant correlation ('*'). (**f**) *SNPs* were accumulated and tallied across all *CTCF* TFBS, with position 16 harbouring the fewest. (**g**) The *DHS enrichment* at SNVs compared to the positional background is significant (q<0.05; '*') at a few positions (5, 16, +2, and +3). (**h**) The mean predicted IGR *Impact Score* of SNVs on transcription factor binding is generally elevated within the motif region.

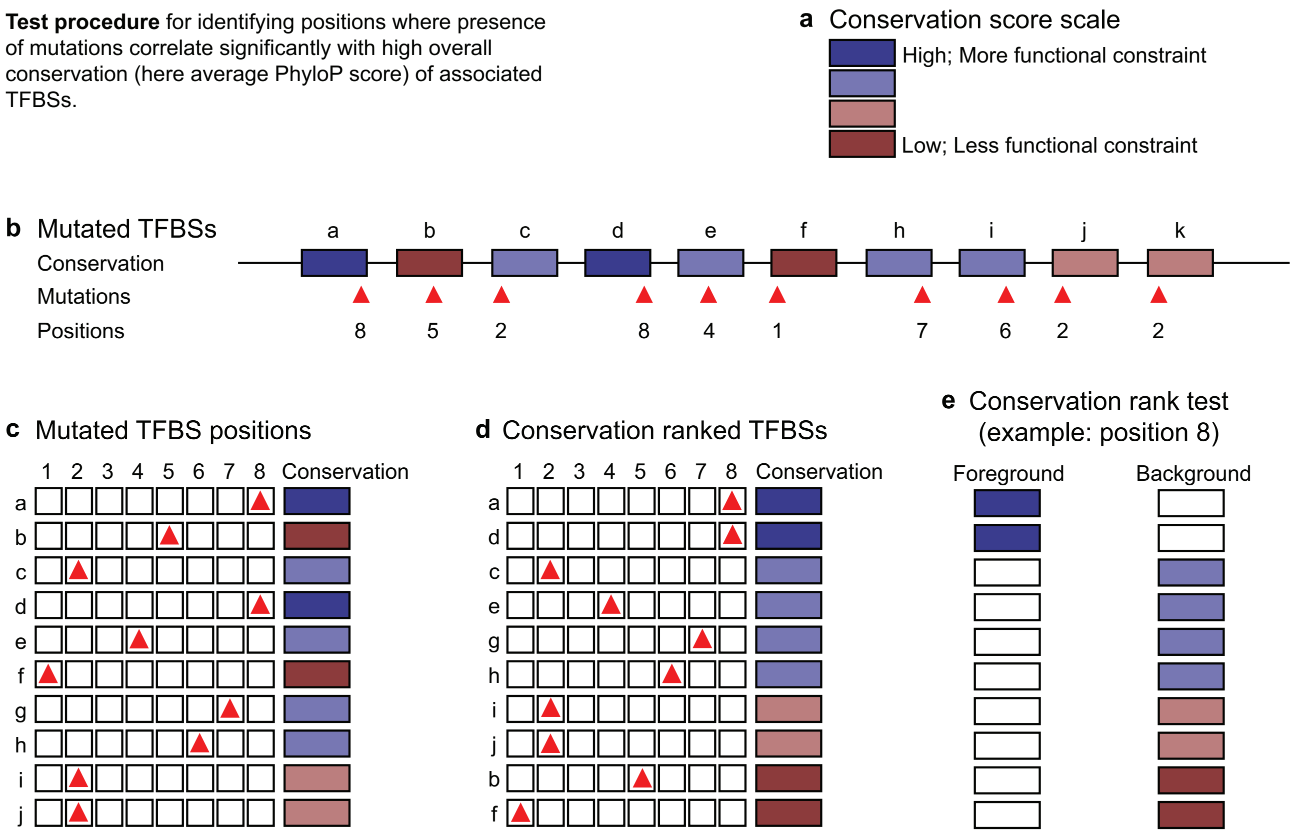

Supplementary Figure 5: Test for correlation between SNV presence and high TFBS conservation. We would like to evaluate if mutations are associated with conserved TFBSs. (**a**) Evolutionary conservation implies functional constraint and in the case of TFBSs, specifically, high binding affinity[3]. For this example, the conservation score is represented by color (blue=high; brown=low). In reality, we use the average PhyloP score across TFBS positions, disregarding mutated positions, as our conservation score. (**b-c**) We would like to apply the test positionally, to evaluate if mutations in some positions of the TFBSs (or in flanking positions) are more associated with conserved TFBS instances than others. Mutations are therefore annotated by which position of the TFBS they hit as well as the overall conservation score of the TFBS instance. (**d**) We use a Wilcoxon rank-sum test to evaluate if TFBS instances mutated at a certain position are more conserved than TFBSs mutated at other positions. Conceptually, we first rank all the TFBSs by their conservation score. (**e**) We thereafter evaluate if the subset of TFBSs mutated at a certain position are surprisingly highly ranked on the list. In this example, the TFBSs mutated at position 8 are highly conserved and would obtain a significant p-value.

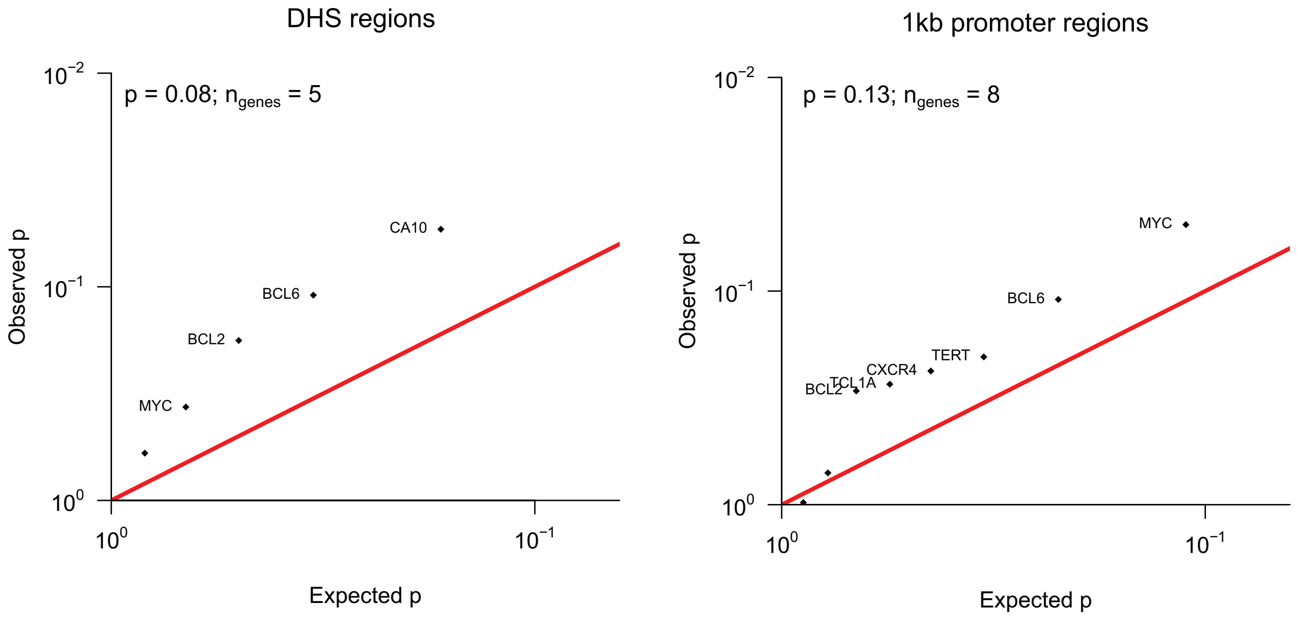

Supplementary Figure 6: Expression correlation of mutations in significant DHS and promoter elements.
(**a**) Gene-expression correlation of mutations (both SNVs and INDELs) in the sets of candidate driver elements of type DHS and (**b**) 1 kb promoters. Rank-sum test p-values of individual genes are shown as qq-plot. Overall significance across all genes is found using Fisher's method and shown in upper left corner. The red line indicates expected p-values under the null hypothesis of no expression correlation.

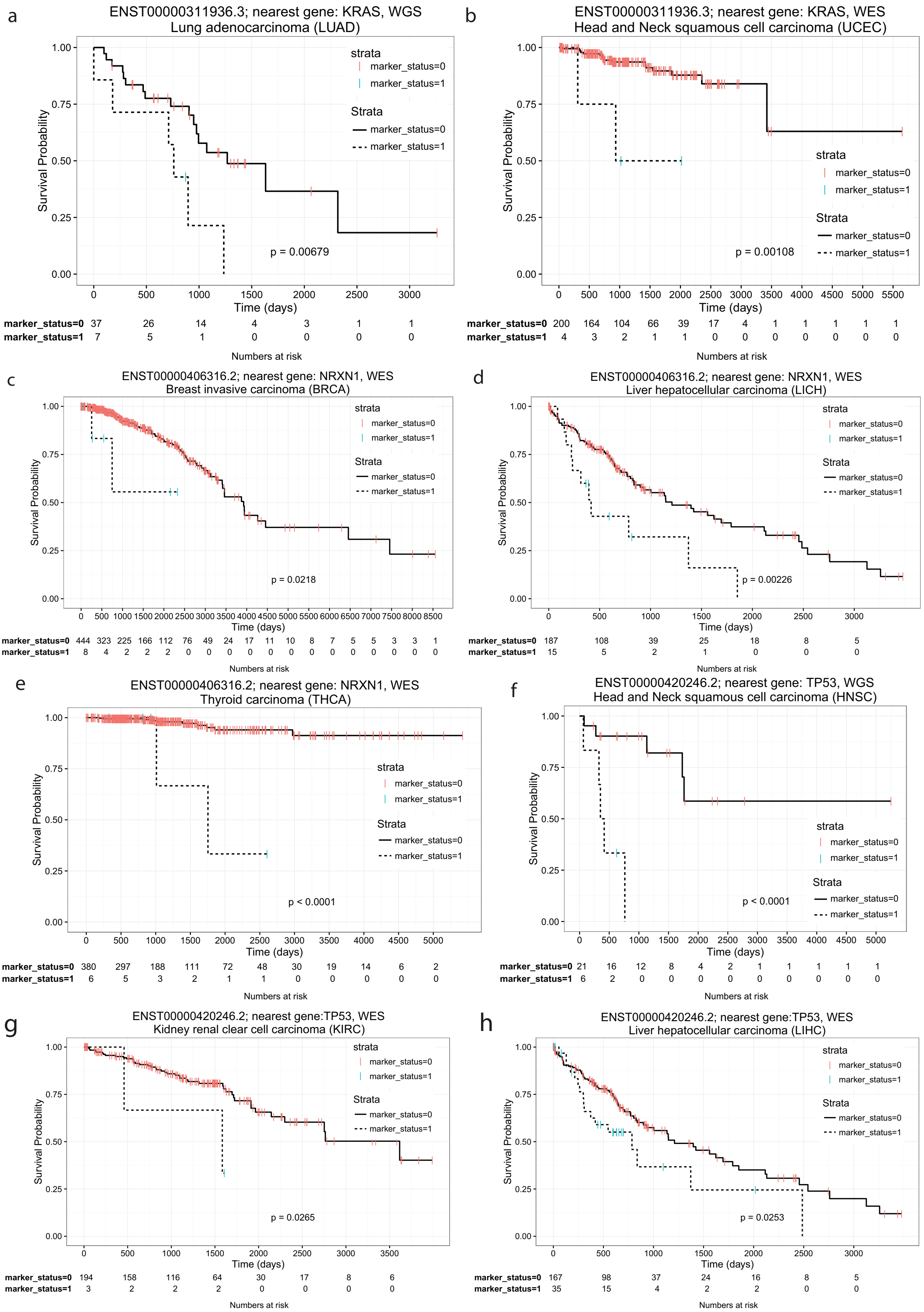

a
ENST00000311936.3; nearest gene: KRAS, WGS
Lung adenocarcinoma (LUAD)
Survival Probability
1.00
0.75
0.50
0.25
0.00
strata
marker_status=0
marker_status=1
Strata
marker_status=0
marker_status=1
p = 0.00679
0 500 1000 1500 2000 2500 3000
Time (days)
marker_status=0 37 26 14 4 3 1 1
marker_status=1 7 5 1 0 0 0 0
Numbers at risk
b
ENST00000311936.3; nearest gene: KRAS, WES
Head and Neck squamous cell carcinoma (UCEC)
Survival Probability
1.00
0.75
0.50
0.25
0.00
strata
marker_status=0
marker_status=1
Strata
marker_status=0
marker_status=1
p = 0.00108
0 500 1000 1500 2000 2500 3000 3500 4000 4500 5000 5500
Time (days)
marker_status=0 200 164 104 66 39 17 4 1 1 1 1 1
marker_status=1 4 3 2 1 1 0 0 0 0 0 0 0
Numbers at risk
c
ENST00000406316.2; nearest gene: NRXN1, WES
Breast invasive carcinoma (BRCA)
Survival Probability
1.00
0.75
0.50
0.25
0.00
strata
marker_status=0
marker_status=1
Strata
marker_status=0
marker_status=1
p = 0.0218
0 500 1000 1500 2000 2500 3000 3500 4000 4500 5000 5500 6000 6500 7000 7500 8000 8500
Time (days)
marker_status=0 444 323 225 166 112 76 49 24 17 11 10 8 7 5 5 3 3 1
marker_status=1 8 4 2 2 2 0 0 0 0 0 0 0 0 0 0 0 0 0
Numbers at risk
d
ENST00000406316.2; nearest gene: NRXN1, WES
Liver hepatocellular carcinoma (LICH)
Survival Probability
1.00
0.75
0.50
0.25
0.00
strata
marker_status=0
marker_status=1
Strata
marker_status=0
marker_status=1
p = 0.00226
0 500 1000 1500 2000 2500 3000
Time (days)
marker_status=0 187 108 39 25 18 8 5
marker_status=1 15 5 2 1 0 0 0
Numbers at risk
e
ENST00000406316.2; nearest gene: NRXN1, WES
Thyroid carcinoma (THCA)
Survival Probability
1.00
0.75
0.50
0.25
0.00
strata
marker_status=0
marker_status=1
Strata
marker_status=0
marker_status=1
p < 0.0001
0 500 1000 1500 2000 2500 3000 3500 4000 4500 5000
Time (days)
marker_status=0 380 297 188 111 72 48 30 19 14 6 2
marker_status=1 6 5 3 2 1 1 0 0 0 0 0
Numbers at risk
f
ENST00000420246.2; nearest gene: TP53, WGS
Head and Neck squamous cell carcinoma (HNSC)
Survival Probability
1.00
0.75
0.50
0.25
0.00
strata
marker_status=0
marker_status=1
Strata
marker_status=0
marker_status=1
p < 0.0001
0 500 1000 1500 2000 2500 3000 3500 4000 4500 5000
Time (days)
marker_status=0 21 16 12 8 4 2 1 1 1 1 1
marker_status=1 6 2 0 0 0 0 0 0 0 0 0
Numbers at risk
g
ENST00000420246.2; nearest gene:TP53, WES
Kidney renal clear cell carcinoma (KIRC)
Survival Probability
1.00
0.75
0.50
0.25
0.00
strata
marker_status=0
marker_status=1
Strata
marker_status=0
marker_status=1
p = 0.0265
0 500 1000 1500 2000 2500 3000 3500
Time (days)
marker_status=0 194 158 116 64 30 17 8 6
marker_status=1 3 2 2 2 0 0 0 0
Numbers at risk
h
ENST00000420246.2; nearest gene:TP53, WES
Liver hepatocellular carcinoma (LIHC)
Survival Probability
1.00
0.75
0.50
0.25
0.00
strata
marker_status=0
marker_status=1
Strata
marker_status=0
marker_status=1
p = 0.0253
0 500 1000 1500 2000 2500 3000
Time (days)
marker_status=0 167 98 37 24 16 8 5
marker_status=1 35 15 4 2 2 0 0
Numbers at risk

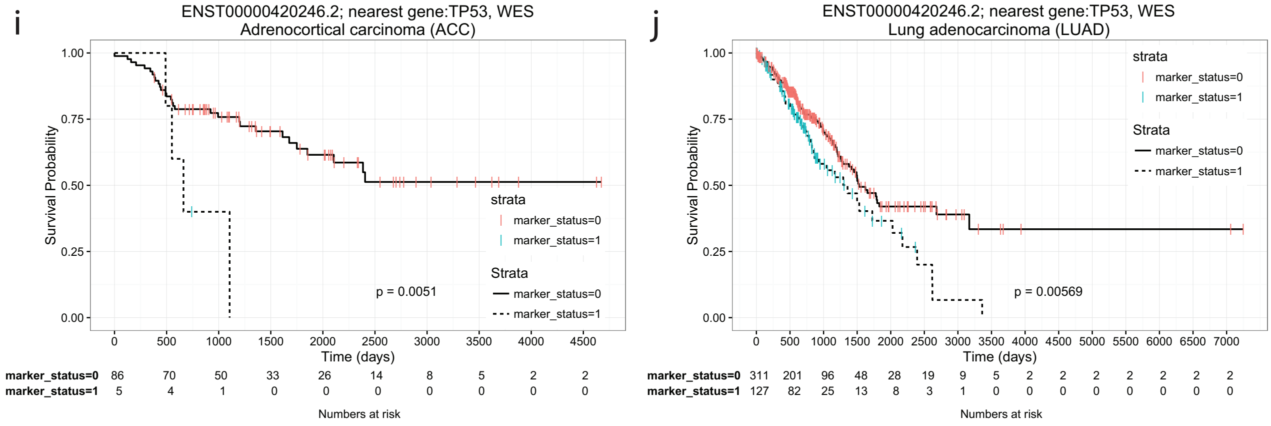

Supplementary Figure 7: Significant cases (p-val < 0.05) of survival correlation with significant protein-coding element mutations.
Cases were only included when candidates were overall significant across cancer types (FDR < 25%). Candidates tested in Whole Genome Sequencing (WGS, see also Supplementary Table 13) and Whole Exome capture Sequencing (WES, see also Supplementary Table 11) cohorts. Coloured tick marks denote patient censoring events. *KRAS* element mutations tested in LUAD (**a**) and UCEC (**b**) cancer types; *NRXN1* element mutations tested in BRCA (**c**), LICH (**d**), and THCA (**e**) cancer types; *TP53* element mutations tested in HNSC (**f**), KIRC (**g**), LIHC (**h**), ACC (**i**), and LUAD (**j**) cancer types.

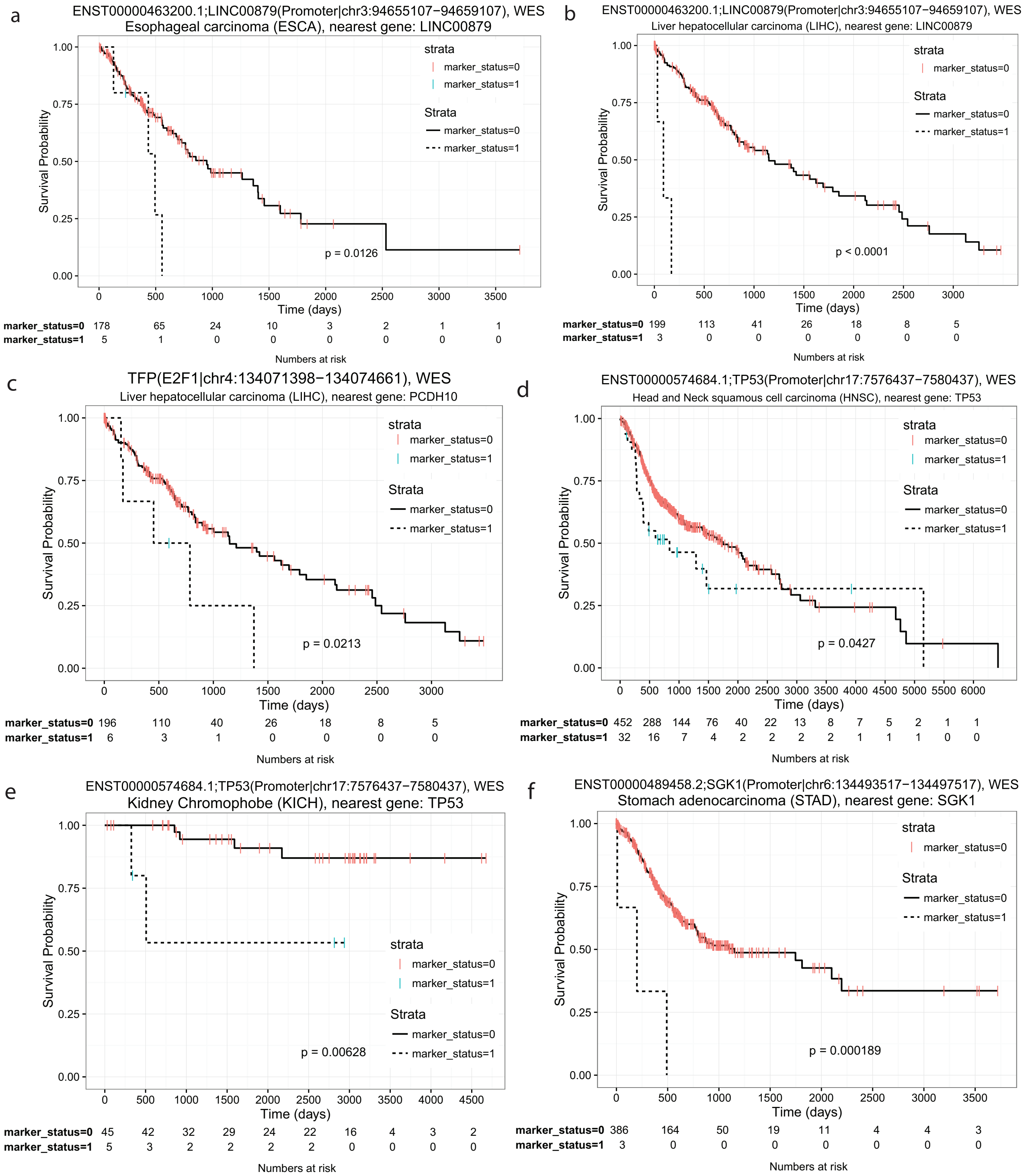

Supplementary Figure 8: Significant cases (p-val < 0.05) of survival correlation with significant non-coding element mutations that were not shown in the main text.
Cases were only included when candidates were overall significant across cancer types (FDR < 25%). Candidates tested in Whole Exome capture Sequencing (WES, see also Supplementary Table 12) cohort. Coloured tick marks denote patient censoring events. *LINC00879* element mutations tested in ESCA (**a**) and LIHC (**b**) cancer types; *PCDH10* element mutations tested in LIHC (**c**) cancer type; *TP53* element mutations tested in HNSC (**d**) and KICH (**e**) cancer types; *SGK1* element mutations tested in STAD (**f**) cancer type.

Supplementary Notes

Supplementary Note 1: Mutation analysis in genome-wide TFBS sets

A large fraction of the individual TFBS elements identified as significant for both SNVs and INDELs are either *CTCF*, *RAD21*, or *SMC3* binding sites (25 of 91; Supplementary Table 2; Fig. 2a.14-21). These three transcription factors are associated with the cohesin complex[4], a key player in formation and maintenance of topological chromatin domains[5,6]. This suggests that non-coding mutations play a role shaping the chromatin structure during cancer development.

Furthermore, it also indicates that binding sites of some transcription factors (TFs) may be overall more perturbed than others in cancer development. To answer this, we screened genome-wide sets of TFBSs corresponding to individual transcription factors. TFBSs for 109 TFs were defined from an ENCODE collection of 1,760,420 genomic sites, in which the TFBS is within a verified TF peak motif region (TPM)[7,8]. Each of the 109 transcription factors may have one or more corresponding binding site motif subtype, for example *CTCF* has 12 binding site subtypes, thus generating a total of 915 TFBS sets. The individual TFBS set was analyzed using the ncDriver procedure to screen for driver mutation evidence (**Fig. 2c**). The ncDriver procedure was applied without the stage 1 mutational recurrence to only evaluate the cancer specificity and conservation properties of the mutations in each TFBS set. This identified transcription factors with significant binding site sets for both SNVs (n=25) and INDELS (n=3; $q<0.05$; **Fig. 2d; Supplementary Table 5**). The TFBSs associated with the cohesin complex (*CTCF*, *RAD21*, and *SMC3*) were top-ranked for both SNVs ($q<1.1\times10^{-7}$) and INDELs ($q<3.4\times10^{-2}$; **Fig. 2d**).

GREAT[9] was used for functional enrichment analysis of significant TFBS sets detected by ncDriver. For each transcription factor, mutated TFBSs were used as forground and the full set of TFBSs as background. The genes associated with the mutated sites are enriched for cancer related terms for the top-ranked TFBS sets (**Supplementary Table 6**).

Supplementary Note 2: Genome-wide mutation analysis of the *CTCF* binding sites

Cohesin binding sites are known to accumulate mutations[10–12], but the mutational mechanism, their potential functional impact, and the potential role of positive selection is not well understood. As the binding sites of the three cohesin-associated transcription factors are largely overlapping, we focused on *CTCF* binding sites (**Supplementary Fig. 4**). More specifically we focused on the subtype disc1 (subtype descriptor 1; disc1; n=23,599), which is the the most common subtype and was the most significant set among the 12 *CTCF* subtypes (**Supplementary Table 5**).

Overall *CTCF* sites are hit 1.8 times more by SNVs than expected by chance ($p=2.5\times10^{-54}$), with the highest enrichment in liver cancer (3.3x) followed by breast cancer (1.5x; **Supplementary Table 5**). In contrast, *CTCF* sites are overall depleted for INDELs (0.7x; $p=9.6\times10^{-1}$). The SNV distribution is uneven along the motif, with a spike in the mutation rate at position 16 (**Supplementary Fig. 4b**). We also find SNV peaks in the 5'-part of the motif and in the 3' flanking region, similar to what was previously reported[10]. Position 15 and 16 are nearly invariant across *CTCF* sites and matches part of the liver-cancer-specific mutational signature 12 (C(T->N)N)[13]. However, the mutation rate remains 18 times higher than expected even when taking the context into account ($p=5.5\times10^{-88}$; **Supplementary Table 7**). To evaluate the context-dependent expected

mutation rate at *CTCF* (subtype disc1) TFBSs in liver cancer, we weighed the occurrence of all possible trinucleotides at the position with their observed mutation rate in the rest of the genome (**Supplementary Table 7**).

We evaluated if mutations at a given position correlated with high overall conservation of the entire TFBS. In short, a Wilcoxon rank-sum test was used to evaluate if instances mutated at a certain position showed higher average phyloP scores than expected (**Supplementary Fig. 5**; **Supplementary Table 8**).

Among *CTCF* sites, position 16 shows the highest evolutionary conservation, signifying its functional importance (**Supplementary Fig. 4d**). The presence of SNVs at position 16 correlates positively with high conservation both for the actual position ($p=2.6 \times 10^{-49}$; Wilcoxon rank-sum test) and site average ($p=3.3 \times 10^{-5}$; **Supplementary Table 8**; **Supplementary Fig. 4e**).

In contrast, the conservation of the 3' flanking region of *CTCF* sites is generally low with SNVs in positions with conservation below average (**Supplementary Fig. 4d**). In contrast, the 3' flanking region is lowly conserved with SNVs that hit positions with below average levels of conservation. However, the *CTCF* binding sites neighboring SNVs in the six bp-long 3' flanking region are surprisingly highly conserved (2.3×10^{-13}; Wilcoxon rank-sum test).

The SNVs in the six bp-long 3' flanking region appear not to impact binding of *CTCF* or other transcription factors (IGR analysis; **Supplementary Fig. 4h**). We used IGR[14] to evaluate the effect of mutations on overall transcription factor binding across the *CTCF* TFBSs. ENCODE ChipSeq and DNaseI signals were used (foldChange scores, March 2012 release)[8]. First, we selected cell types for which DNaseI peaks were available. In the case that there were multiple conditions assayed, the normal or control set of peaks was used, and University of Washington peaks were preferred for DNaseI. Then we took all individual ChipSeq experiment tracks for each cell type and ran the IGR 7-mer and 8-mer models from them separately on all mutations. Finally, the models associated with the same factor were summed across cell types and conditions to reduce potential noise or cell type specific conditions not preserved in the aberrantly expressive cancer cells. A final overall IGR impact score was associated with each position.

A low SNP frequency at the position signifies high levels of purifying selection or potentially low rates of mutation (**Supplementary Fig. 4f**). For the SNP and INDEL polymorphism count profiles across the *CTCF* TFBSs, we downloaded dbSNP build 138 (ftp.ncbi.nih.gov/snp). We used the dbSNP class annotation to divide into SNPs (class: 'single') and INDELs (classes: 'in-del', 'insertion' or 'deletion'). These were intersected with the *CTCF* TFBSs and their 30 bp flanking regions.

For the analysis of DHS elements, we mapped each cancer type to its closest normal Epigenomic Roadmap tissue[15] (**Supplementary Table 9**) and extracted the corresponding imputed DNaseI tracks[16]. Using these, TFBSs and their flanking regions were annotated as being open (DHS) or closed for each cancer type. Finally, the enrichment in DHS level between mutated and non-mutated positions were aggregated across tissues and associated with a Fisher's exact test p-value (**Supplementary Fig. 4g**, DHS enrichment). This demonstrated, that SNVs are enriched for overlap with DNase hypersensitive sites, signifying a higher mutation rate in regions with open chromatin and likely *CTCF* occupancy (**Supplementary Fig. 4g**).

Together, these observations show that the mutation rate is elevated at highly conserved and high affinity *CTCF* binding sites in active, open-chromatin regions[17]. The increase in mutation rate not only at functionally important sites (position 16), but also at apparently non-functional sites (3'

flanking region), suggests that much of the increase may be driven by mutational mechanisms caused by micro-environment conditions coupled to *CTCF* binding. Specifically, spacer DNA regions between the core *CTCF* binding site and flanking optional binding sites appear to be physically bent during binding[18,19], which may affect mutation rates.

Supplementary References

1. Parker, B. J. *et al.* New families of human regulatory RNA structures identified by comparative analysis of vertebrate genomes. *Genome Res.* **21,** 1929–1943 (2011).
2. Darty, K., Denise, A. & Ponty, Y. VARNA: Interactive drawing and editing of the RNA secondary structure. *Bioinformatics* **25,** 1974–1975 (2009).
3. Jaeger, S. A. *et al.* Conservation and regulatory associations of a wide affinity range of mouse transcription factor binding sites. *Genomics* **95,** 185–195 (2010).
4. Hou, C., Dale, R. & Dean, A. Cell type specificity of chromatin organization mediated by CTCF and cohesin. *Proc. Natl. Acad. Sci. U. S. A.* **107,** 3651–3656 (2010).
5. Zuin, J. *et al.* Cohesin and CTCF differentially affect chromatin architecture and gene expression in human cells. *Proc. Natl. Acad. Sci. U. S. A.* **111,** 996–1001 (2014).
6. Sanborn, A. L. *et al.* Chromatin extrusion explains key features of loop and domain formation in wild-type and engineered genomes. *Proc. Natl. Acad. Sci. U. S. A.* **112,** E6456–65 (2015).
7. Khurana, E. *et al.* Integrative annotation of variants from 1092 humans: application to cancer genomics. *Science* **342,** 1235587 (2013).
8. Bernstein, B. E. *et al.* An integrated encyclopedia of DNA elements in the human genome. *Nature* **489,** 57–74 (2012).
9. McLean, C. Y. *et al.* GREAT improves functional interpretation of cis-regulatory regions. *Nat. Biotechnol.* **28,** 495–501 (2010).
10. Katainen, R. *et al.* CTCF/cohesin-binding sites are frequently mutated in cancer. *Nat. Genet.* **47,** 818–821 (2015).

11. Dai, J. *et al.* Systematical analyses of variants in CTCF-binding sites identified a novel lung cancer susceptibility locus among Chinese population. *Sci. Rep.* **5,** 7833 (2015).

12. Umer, H. M. *et al.* A Significant Regulatory Mutation Burden at a High-Affinity Position of the CTCF Motif in Gastrointestinal Cancers. *Hum. Mutat.* **37,** 904–913 (2016).

13. Alexandrov, L. B. *et al.* Signatures of mutational processes in human cancer. *Nature* **500,** 415–421 (2013).

14. Cowper-Sal lari, R. *et al.* Breast cancer risk-associated SNPs modulate the affinity of chromatin for FOXA1 and alter gene expression. *Nat. Genet.* **44,** 1191–1198 (2012).

15. Polak, P. *et al.* Cell-of-origin chromatin organization shapes the mutational landscape of cancer. *Nature* **518,** 360–364 (2015).

16. Roadmap Epigenomics Consortium *et al.* Integrative analysis of 111 reference human epigenomes. *Nature* **518,** 317–330 (2015).

17. Jaeger, S. A. *et al.* Conservation and regulatory associations of a wide affinity range of mouse transcription factor binding sites. *Genomics* **95,** 185–195 (2010).

18. MacPherson, M. J. & Sadowski, P. D. The CTCF insulator protein forms an unusual DNA structure. *BMC Mol. Biol.* **11,** 101 (2010).

19. Nakahashi, H. *et al.* A genome-wide map of CTCF multivalency redefines the CTCF code. *Cell Rep.* **3,** 1678–1689 (2013).

13 HYPERLINK "http://paperpile.com/b/SEoPai/tBg3"
